# A post-hoc internal validation of arginine-stimulated copeptin cut-offs for diagnosing AVP deficiency (central diabetes insipidus)

**DOI:** 10.1007/s11102-025-01523-2

**Published:** 2025-04-26

**Authors:** Cihan Atila, Bettina Winzeler, Irina Chifu, Martin Fassnacht, Julie Refardt, Mirjam Christ-Crain

**Affiliations:** 1 Department of Endocrinology, Diabetology and Metabolism, University Hospital Basel, Basel, Switzerland; 2 Department of Clinical Research, University Hospital Basel, University of Basel, Basel, Switzerland; 3 Department of Internal Medicine, Section of Endocrinology, Erasmus Medical Centre, Rotterdam, The Netherlands; 4 Division of Endocrinology and Diabetes, Department of Internal Medicine I, University Hospital, University of Wuerzburg, Wuerzburg, Germany; 5 Central Laboratory, University Hospital Wuerzburg, Wuerzburg, Germany; 6 Department of Endocrinology, Diabetes and Metabolism, University Hospital Basel, Petersgraben 4, Basel, 4031 Switzerland

**Keywords:** Copeptin, Sodium, Osmolality, Primary polydipsia, Polyuria, Polydipsia, Polyuria polydipsia syndrome, Hypertonic saline, Arginine test, AVP, Diagnostic, Algorithm

## Abstract

**Background:**

Distinguishing arginine vasopressin (AVP) deficiency (central diabetes insipidus) from primary polydipsia is challenging. While hypertonic saline-stimulated copeptin testing provides the highest diagnostic accuracy, it is often restricted to specialised centres, requiring close monitoring and potentially causing patient discomfort. Initially, arginine-stimulated copeptin was proposed as a simpler alternative, but a head-to-head comparison study found it less precise than hypertonic saline stimulation. However, the same study identified two new high sensitivity and specificity cut-offs for arginine-stimulated copeptin, though these cut-offs have yet to be validated.

**Methods:**

This is a secondary post-hoc analysis of the initial prospective multicentre study, including adult patients with confirmed AVP deficiency or primary polydipsia. Participants underwent the arginine stimulation test, with plasma copeptin measured at baseline and 60- and 90 min after arginine infusion. The primary objective was to revisit the original study to internally validate the proposed arginine-stimulated copeptin cut-offs of > 5.2pmol/L (high specificity cut-off with > 90% specificity for primary polydipsia) and ≤ 3.0 pmol/L (high specificity cut-off with > 90% specificity for AVP deficiency).

**Findings:**

In total, 96 patients were included between May 2013 and June 2018: *n* = 38 [40%] with AVP deficiency and *n* = 58 [60%] with primary polydipsia. At 60 min after arginine infusion, a copeptin level ≤ 3.0 pmol/L showed a specificity of 95% (95% CI: 0.88-1.00) for AVP deficiency, while a copeptin level > 5.2 pmol/L demonstrated a specificity of 97% (95% CI: 0.92-1.00) for primary polydipsia. The ≤ 3.0 pmol/L cut-off accurately identified 71% (*n* = 27/38) of patients with AVP deficiency, and the > 5.2 pmol/L cut-off correctly identified 69% (*n* = 40/58) of patients with primary polydipsia.

**Interpretation:**

This analysis validates two new copeptin cut-offs of the arginine stimulation test to distinguish AVP deficiency from primary polydipsia: >5.2 pmol/L for high specificity in diagnosing primary polydipsia and ≤ 3.0 pmol/L for high specificity in diagnosing AVP deficiency. These thresholds might offer a practical initial alternative to hypertonic saline testing.

**Registration:**

Clinicaltrials.gov (NCT00757276).

## Introduction

Disruptions in the hypothalamic-posterior pituitary axis can result in arginine vasopressin (AVP) deficiency (central diabetes insipidus), clinically manifesting as hypotonic polyuria and polydipsia [1–3]. The main differential diagnosis is primary polydipsia, characterised by excessive fluid intake despite adequate AVP secretion or renal function [1–3]. Accurate differentiation between these conditions is crucial due to the distinct treatment strategies required [4–6].

The development of copeptin as a reliable and stable marker for AVP has introduced direct testing approaches with high diagnostic accuracy [7–11]. Over the past decade, copeptin-based testing has demonstrated robust performance, with hypertonic saline-stimulated copeptin achieving over 95% diagnostic accuracy and arginine-stimulated copeptin achieving an initial accuracy of 93% [1, 12–16]. While the hypertonic saline test is highly accurate, it requires specialised settings with continuous monitoring and rapid sodium measurements, making it impractical for widespread use. Conversely, arginine stimulation, widely used in anterior pituitary evaluations, is simpler to perform and familiar to most clinicians.

In 2023, a direct, multicentre head-to-head comparison study showed arginine-stimulated copeptin with a best cut-off of 3.8 pmol/L to be less precise than hypertonic saline for diagnosing AVP deficiency with an accuracy of only 74% [12]. Yet, stimulated copeptin levels at 60 min of 3.0 pmol/L or below and above 5.2 pmol/L in arginine testing showed > 90% specificity and sensitivity, accurately diagnosing over half of the patients [12]. Given these findings, arginine stimulation was recommended as an initial, well-tolerated diagnostic test, especially where hypertonic saline is unavailable or contraindicated. However, these proposed new cut-offs have not been validated so far. Therefore, this secondary analysis aims to revisit the original study [16] to validate the proposed arginine-stimulated copeptin cut-offs and assess their diagnostic utility.

## Materials and methods

### Study design

This secondary post-hoc analysis of a prospective diagnostic study assessed the arginine stimulation test for diagnosing patients with hypotonic polyuria-polydipsia syndrome [16]. The study, conducted between May 2013 and June 2018, involved five recruiting centres in Switzerland (Basel, Aarau, Lucerne, Bern, and St. Gallen) and the University Hospital Würzburg in Germany. All tests were performed in Basel, Switzerland. The study received approval from the local ethics committees of all participating centres, and written informed consent was obtained from all patients before inclusion. The study was preregistered on Clinicaltrials.gov (NCT00757276).

### Study participants and procedures

Adult patients with a previously confirmed diagnosis of AVP deficiency or primary polydipsia were recruited. A standardised baseline visit evaluated symptoms and concomitant diseases at inclusion. For the main study visit, participants presented at site in the morning after an overnight meal fasting. They were permitted to drink water until 6 A.M, i.e., two hours before the first baseline blood sample was taken. Patients under desmopressin treatment were instructed to cease the medication 24 h before testing. Patients on hydrocortisone therapy received an individualised stress dose. Participants were administered venous access and an infusion of 0.5 g per kg body weight L-Arginine Hydrochloride (21%) diluted in 500 ml 0.9% normal saline was given over 30 min. Blood samples were collected before, 60 and 90 min after starting the infusion.

According to the study in 2023 [12], the new suggested copeptin cut-offs at 60 min after arginine infusion were:

- Stimulated plasma copeptin levels of ≤ 3.0 pmol/L: >90% specificity for AVP deficiency.
- Stimulated plasma copeptin levels of > 5.2 pmol/L: >90% specificity for primary polydipsia.

The new suggested copeptin cut-offs at 90 min after arginine infusion were:

- Stimulated plasma copeptin levels of ≤ 3.0 pmol/L: >90% specificity for AVP deficiency.
- Stimulated plasma copeptin levels of > 6.0 pmol/L: >90% specificity for primary polydipsia.

Patients from this study, were not included in the subsequent head-to-head CARGOx trial [12], essential to allow for proper validation of the initially proposed cut-offs in the later head-to-head trial.

### Laboratory measurements

Samples were taken as aliquots, immediately centrifuged at 4 °C at 1500 × g for 10 min, then stored at < − 70 °C until batch analysis. All copeptin measurements were performed using the BRAHMS Copeptin proAVP assay (Thermo Scientific Biomarkers, Hennigsdorf, Germany), a CE-certified automated immunoassay. The lower detection limit was 0.4 pmol/L, the inter-assay coefficient of variation was 7.0%, and the intra-assay coefficient of variation was 9.8%. The glomerular filtration rate (GFR) was estimated using the CKD-EPI equation, based on serum creatinine, age, and sex, and standardized to a body surface area of 1.73 m^2^ [17].

### Statistical analysis

This primary aim was to revisit the original study [16] and validate the newly proposed arginine-stimulated copeptin cut-offs at 60 min and 90 min after arginine stimulation. Demographic information, laboratory parameters, and test tolerability were described using median [IQR] or absolute (relative) frequency as appropriate. Sensitivity and specificity with a 95% Wilson confidence interval (CI) are indicated as diagnostic measures for each copeptin cut-off for both time points. We performed the following sensitivity analysis: First, the analysis was repeated only in patients who additionally underwent the hypertonic saline test, followed by the arginine stimulation test (*n* = 60 out of 96). Second, excluding patients with severe nausea during the test, which could potentially result in higher copeptin values. Nausea was rated on a numeric rating scale at all timepoints (0 = no symptom, 10 = maximum symptom). We performed a sensitivity analysis and excluded all patients reporting nausea greater than 5 points, either at 60–90 min and/or vomiting (*n* = 7 patients excluded). All analyses were conducted using the statistical software package R (version 4.2.3) [18].

## Results

### Baseline characteristics

A total of 96 patients underwent the arginine stimulation test from May 2013 to September 2018; 38 (40%) had AVP deficiency, and 58 (60%) had primary polydipsia. The median age for patients with AVP deficiency was 43 years [IQR, 31–54], with 58% female, compared to a median age of 35 years [30–41] and 72% female for patients with primary polydipsia. Among patients with AVP deficiency, 17 (45%) exhibited isolated posterior pituitary dysfunction, and 21 (55%) had combined anterior and posterior pituitary dysfunction. Patients with AVP deficiency and primary polydipsia showed similar total volumes of polyuria and polydipsia, baseline plasma sodium, and plasma copeptin levels. Baseline characteristics are summarised in Table 1.

**Table 1:** Baseline characteristics

	AVP Deficiency	Primary Polydipsia
	*n* = 38	*n* = 58
Female sex	22 (58)	42 (72)
Age, years	43 [31, 54]	35 [30, 41]
Body-mass index†	26.7 (5.4)	25.0 (5.6)
Caucasian ethnicity	36 (95)	55 (95)
**Clinical symptoms**		
Polydipsia, ml consumed/day	5500 [4000, 7000]	7000 [5000, 8000]
Fluid intake at night	30 (79)	49 (84)
**Laboratory data**		
Plasma sodium, mmol/litre	142 [140, 144]	140 [139, 142]
Plasma osmolality, mOsm/kg	292 [289, 296]	291 [289, 295]
Plasma copeptin, pmol/litre	2.1 [1.9, 2.7]	3.6 [2.4, 5.7]
Estimated GFR (ml/min/1.73m^2^)	94 [83, 103]	102 [87, 114]
**Medical history**		
Previous pituitary surgery	18 (47)	2 (3)
Anterior pituitary deficiency	21 (55)	2 (3)

† The body-mass index is the weight in kilograms divided by the square of the height in meters. GFR glomerular filtration rate. Data are presented in median [IQR] or no. (%)

### Stimulated copeptin after arginine infusion

**Fig. 1:**
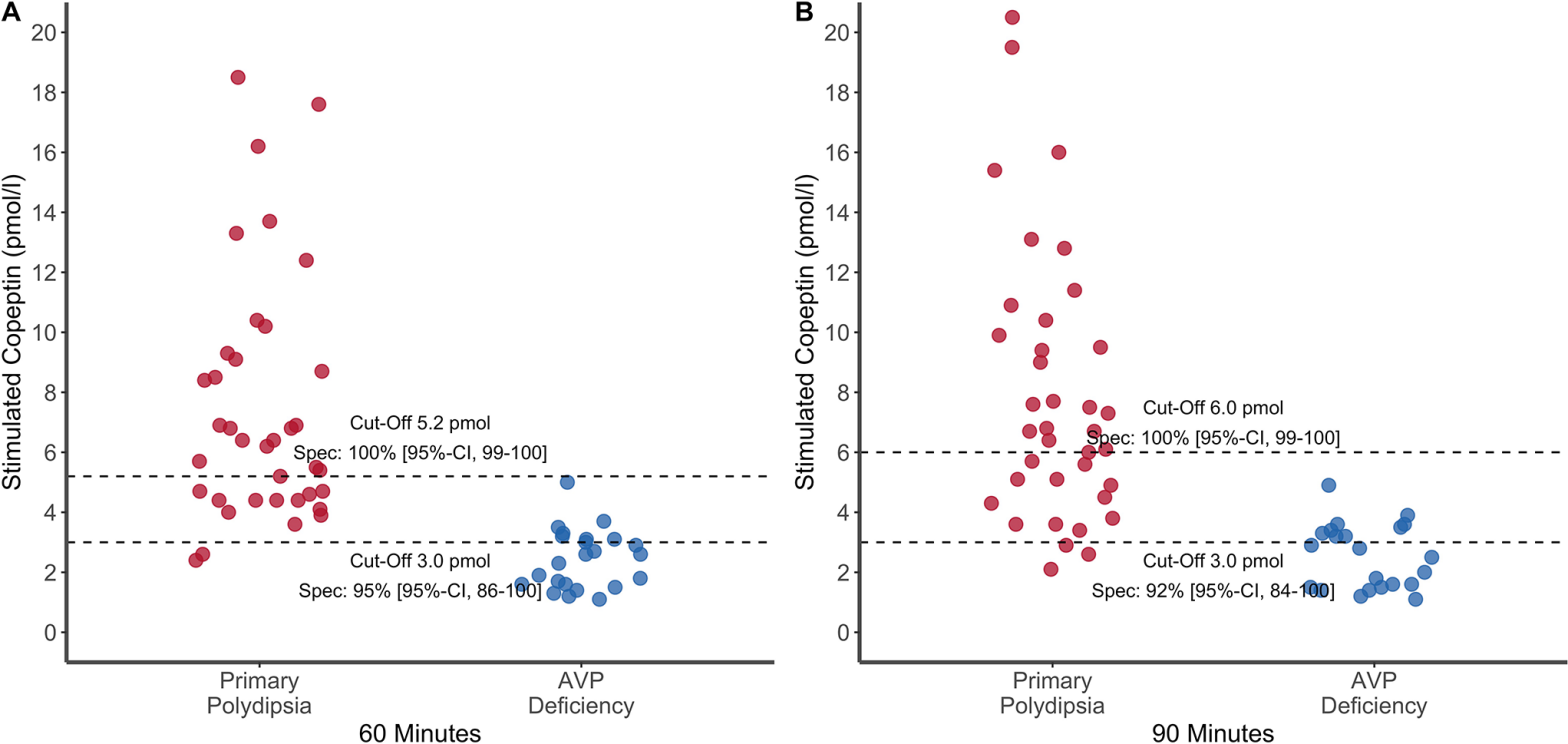
Stimulated plasma copeptin. Stimulated plasma copeptin after arginine infusion at (**A**) 60 min and (**B**) 90 min. Data are expressed as individual points for each patient, for patients with arginine vasopressin deficiency (in blue) and primary polydipsia (in red). The dashed lines represent the > 90%-specificity and > 90%-sensitivity cutoff derived from *Refardt J et al. 2023* [12]

For both time points, the stimulated copeptin after arginine infusion is shown in Fig. 1.

As shown in the original study [16], at 60 min, the overall best cut-off of 3.8 pmol/l provided a sensitivity of 93% (95% CI [0.86–0.98]) and specificity of 92% (95% CI [0.86–0.97]), correctly diagnosing 93% (*n* = 54/58) of patients with primary polydipsia and 92% (*n* = 35/38) with AVP deficiency. At 60 min, a stimulated copeptin of > 5.2 pmol/l provided a specificity of 97% (95% CI [0.92-1.00]) and sensitivity of 69% (95% CI [0.57–0.81]) for diagnosing primary polydipsia, correctly diagnosing 69% (*n* = 40/58) of patients with primary polydipsia and falsely diagnosing 3% (*n* = 1/38) patients with AVP deficiency. At 60 min, a stimulated copeptin of ≤ 3.0 pmol/l provided a sensitivity of 71% (95% CI [0.55–0.84]) and specificity of 95% (95% CI [0.88-1.00]) for diagnosing AVP deficiency, falsely diagnosing 5% (*n* = 3/58) of patients with primary polydipsia and correctly diagnosing 71% (*n* = 27/38) patients with AVP deficiency. In 26% (*n* = 25/96) of patients, stimulated plasma copeptin at 60 min was inconclusive between both cut-offs.

Similarly, as shown in the original study [16], at 90 min, the overall best cut-off of 4.1 pmol/l provided a sensitivity of 86% (95% CI [0.77–0.95]) and specificity of 94% (95% CI [0.86-1.00]), correctly diagnosing 84% (*n* = 49/58) of patients with primary polydipsia and 89% (*n* = 34/38) with AVP deficiency. At 90 min, a stimulated copeptin of > 6.0 pmol/l provided a specificity of 97% (95% CI [0.92-1.00]) and sensitivity of 65% (95% CI [0.53–0.77]) for diagnosing primary polydipsia, correctly diagnosing 64% (*n* = 37/58) of patients with primary polydipsia and falsely diagnosing 3% (*n* = 1/38) patients with AVP deficiency. At 90 min, a stimulated copeptin of ≤ 3.0 pmol/l provided a sensitivity of 64% (95% CI [0.47–0.78]) and specificity of 95% (95% CI [0.88-1.00]) for diagnosing AVP deficiency, falsely diagnosing 5% (*n* = 3/58) of patients with primary polydipsia and correctly diagnosing 61% (*n* = 23/38) patients with AVP deficiency. In 30% (*n* = 29/96) of patients, stimulated plasma copeptin at 90 min was inconclusive between both cut-offs.

### Sensitivity analysis

**Fig. 2:**
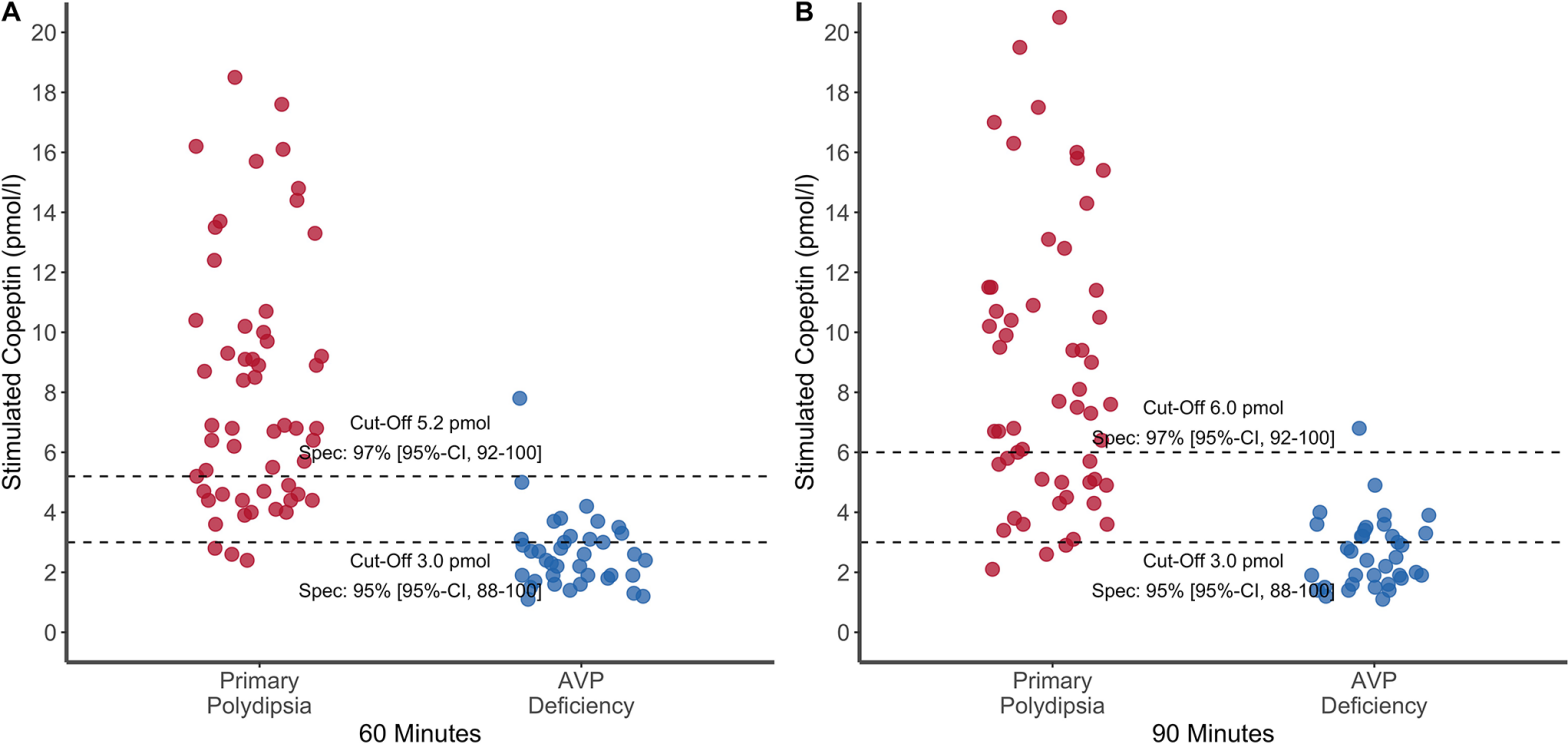
Sensitivity analysis of stimulated plasma copeptin. Sensitivity analysis only in patients who additionally underwent the hypertonic saline test first, followed by the arginine stimulation test (*n* = 60 out of 96). Stimulated plasma copeptin after arginine infusion at (**A**) 60 min and (**B**) 90 min. Data are expressed as individual points for each patient, for patients with arginine vasopressin deficiency (in blue) and primary polydipsia (in red). The dashed lines represent the > 90%-specificity and > 90%-sensitivity cutoff derived from *Refardt J et al. 2023* [12]

For both time points, the sensitivity analysis of stimulated copeptin after arginine infusion is shown in Fig. 2. In the subset of patients undergoing additional hypertonic saline stimulation test (*n* = 60 out of 96), at 60 min, a stimulated copeptin of > 5.2 pmol/l provided a specificity of 100% (95% CI [0.99-1.00]) for diagnosing primary polydipsia and a stimulated copeptin of ≤ 3.0 pmol/l provided a specificity of 95% (95% CI [0.86-1.00]) for diagnosing AVP deficiency. At 90 min, a stimulated copeptin of > 6.0 pmol/l provided a specificity of 100% (95% CI [0.99-1.00]) for diagnosing primary polydipsia and a stimulated copeptin of ≤ 3.0 pmol/l provided a specificity of 92% (95% CI [0.84-1.00]) for diagnosing AVP deficiency.

In the subset of patients without relevant nausea (*n* = 89 out of 96), at 60 min, a stimulated copeptin of > 5.2 pmol/l provided a specificity of 97% (95% CI [0.91-1.00]) for diagnosing primary polydipsia and a stimulated copeptin of ≤ 3.0 pmol/l provided a specificity of 95% (95% CI [0.88-1.00]) for diagnosing AVP deficiency. At 90 min, a stimulated copeptin of > 6.0 pmol/l provided a specificity of 97% (95% CI [0.92-1.00]) for diagnosing primary polydipsia and a stimulated copeptin of ≤ 3.0 pmol/l provided a specificity of 95% (95% CI [0.88-1.00]) for diagnosing AVP deficiency.

## Discussion

This secondary analysis validates newly proposed copeptin cut-offs after arginine infusion for diagnosing AVP deficiency. Specifically, at 60 min, a stimulated copeptin level of > 5.2 pmol/L shows a high specificity in diagnosing primary polydipsia and a stimulated copeptin of ≤ 3.0 pmol/L a high specificity in diagnosing AVP deficiency. Applying these cut-offs correctly identified 69% of patients with primary polydipsia and 71% of patients with AVP deficiency.

Studies reveal substantial delays in the initial diagnostic work-up in suspected cases of AVP deficiency, with the average time from symptom onset to final diagnosis of up to 12 months, partly explained by unfamiliarity with the diagnostic work-up and lack of accurate test methods for the outpatient setting [19–22]. Although some clinical symptoms (e.g., large amounts of night time fluid intake or sudden onset of polyuria and polydipsia), medical histories (e.g., prior history of pituitary surgery), and basal laboratory parameters (e.g., elevated basal plasma sodium) can hint at AVP deficiency already during the initial consultation, these parameters have not yet been validated and therefore dynamic testing is often performed. For instance, the classic indirect water deprivation test, traditionally regarded as the gold standard, shows only around 70% accuracy, often necessitating multiple rounds of testing and further extending diagnostic delays [15]. Currently, the copeptin-based hypertonic saline test is the most accurate method for diagnosing AVP deficiency [11, 12, 15]. However, it presents notable challenges: the test requires dual-arm venous access and rapid sodium monitoring to monitor hypernatremia, which confines its feasibility to settings equipped with specific monitoring capabilities. Conversely, arginine stimulation testing offers a simpler, safer, and better-tolerated option for initial diagnosis than hypertonic saline stimulation [16]. Most clinicians are familiar with its protocol, which can be performed in the outpatient setting [23–26]. While it lacks a single optimal copeptin cut-off, the head-to-head comparison in 2023 demonstrated that the threshold values of ≤ 3.0 pmol/L exhibit > 90% specificity for identifying AVP deficiency and > 5.2 pmol/L > 90% specificity for primary polydipsia. These cut-offs enabled the identification of ∼ 70% of patients with AVP deficiency and primary polydipsia [12]. Interestingly, our results validating these cutoffs in the original study demonstrate an even higher specificity of 95% and a sensitivity of 97% of the proposed cut-offs, potentially due to higher copeptin levels observed in patients with primary polydipsia than in the head-to-head study with more severe cases of primary polydipsia. Applying these cut-offs accurately also identified ∼ 70% of patients with AVP deficiency and primary polydipsia in this current analysis. It is important to mention the test duration of 60 min for the arginine stimulation compared to the > 16 h for the classic indirect water deprivation test [15]. Accordingly, the arginine stimulation can be recommended as a simple and well-tolerated initial test in centres where the hypertonic saline test cannot be performed or in patients where it is contraindicated such as in patients with epilepsy, poorly controlled hypertension or heart failure. Patients with copeptin levels between the above cut-offs (30% in this present validation study) or patients experiencing severe nausea or vomiting during the arginine stimulation should, however, undergo further testing. Importantly, the proposed copeptin cut-offs should be applied strictly within the context of the published test protocol in regard to the volume and infusion rate [16]. Variations in either volume or infusion rate may lead to different copeptin levels and impact diagnostic accuracy.

The strength of this study is its well-characterized cohort of nearly 100 patients. However, we acknowledge that the study mainly included patients with mild forms of primary polydipsia and cut-offs should, therefore, be assessed in an external larger validation cohort of more severe cases of primary polydipsia– ideally in a real-word setting. Furthermore, it is crucial to measure plasma copeptin in a non-stressed state, avoiding recent exercise or illness, and consider interfering medications that might influence AVP release or action. Additionally, impaired kidney function can influence plasma copeptin and sodium levels, making GFR assessment important for accurate interpretation. In this cohort, no patients with chronic kidney disease were included, and therefore, the proposed cut-offs should not be applied to patients with impaired kidney function. Since inflammation (e.g., IL-6) can stimulate AVP release, measuring CRP as a marker of inflammation should also be considered [27]. Furthermore, like in previous studies, the main limitation of this study is the absence of a clear diagnostic standard for AVP deficiency. While the diagnoses for our cohort were based on a careful review of the patient data, including laboratory and imaging results and response to desmopressin treatment, not all patients had the hypertonic saline testing as the current gold standard. To mitigate incorporation bias, both trials integrated the treatment response at 3 months into the final diagnosis. Finally, the maximum L-arginine dose of 40 g used in the subsequent head-to-head trial should be mentioned as a potential explanation of lower stimulated copeptin levels in comparison to this study.

In conclusion, our analysis validates the newly suggested copeptin cut-offs for the arginine stimulation test. This simpler testing method can be used in a broader range of clinical settings, enhancing accessibility and reducing patient discomfort as an initial alternative to hypertonic saline testing. As smaller studies have explored the arginine stimulation test in paediatric patients, showing promising results, the arginine stimulation test would also be highly important for this patient population and should be assessed in further studies, also together with alternative promising copeptin-stimulation tests such as glucagon or urea [28–33].

## Data Availability

No datasets were generated or analysed during the current study.
